# Autochthonous *Bacillus licheniformis*: Probiotic potential and survival ability in low‐fishmeal extruded pellet aquafeed

**DOI:** 10.1002/mbo3.767

**Published:** 2018-11-16

**Authors:** Kai‐Min Niu, Damini Kothari, Woo‐Do Lee, Jeong‐Min Lim, Sanaz Khosravi, Sang‐Min Lee, Bong‐Joo Lee, Kang‐Woong Kim, Hyon‐Sob Han, Soo‐Ki Kim

**Affiliations:** ^1^ Department of Animal Biotechnology Konkuk University Seoul Korea; ^2^ Department of Animal Science and Technology Konkuk University Seoul Korea; ^3^ Department of Marine Biotechnology Gangneung Wonju National University Gangneung Korea; ^4^ Aquafeed Research Center National Institute of Fisheries Science Pohang Korea; ^5^ Department of Marine Life and Applied Sciences Kunsan National University Kunsan Korea

**Keywords:** *Bacillus licheniformis*, low‐fishmeal aquafeed, olive flounder, probiotics, survival ability

## Abstract

In recent years, *Bacillus* spp. have garnered attention as probiotic supplements in aquafeed owing to the production of heat stable and low pH resistant spores. Herein, we isolated and characterized an autochthonous *Bacillus licheniformis* KCCM 43270 from the intestine of olive flounder (*Paralichthys olivaceus*) for supplementation in low‐fishmeal extruded aquafeeds. The KCCM 43270 was screened based on amylase, protease, cellulase, and lipase as well as non‐hemolytic activities. The isolate was able to grow in carboxymethyl cellulose (CMC), xylan, and soybean meal (SBM) when used as a single carbon source in the minimal nutrient M9 medium. The KCCM 43270 spores displayed complete survival in acid (pH 2.5) and bile (0.3%, w/v) for 3 hr, strong biofilm formation, and nearly 50% adhesion with intestinal mucus. The spores of the isolate also showed significant survival ability at 80, 90, 100°C for 60, 30, and 1 min, respectively. In addition, the spores in a blend of SBM complex carrier showed significant heat stability at 120°C for 5 min and under different drying conditions. Furthermore, the spores also survived the extrusion process during low‐fishmeal aquafeed manufacturing, implying the potential application of *B. licheniformis* KCCM 43270 in aquafeed industry.

## 1 INTRODUCTION

Olive flounder (*Paralichthys olivaceus*), also known as Japanese flounder is the most economically important marine carnivorous species cultured in Republic of Korea. This species requires high‐protein content diet (~40%–50%) for its optimum growth and performance. Fish meal (FM; 60%–72% crude protein) is the most expensive component in fish feed formulations, accounting for approximately US$ 6 billion in 2017 (https://www.futuremarketinsights.com/reports/fish-meal-market). To attain sustainable aquaculture production, the aquafeed industry must reduce the dependence upon FM by the inclusion of alternative protein sources in feed. Soybean meal (SBM; 40%–51% crude protein) with a balanced amino acid profile as well as low cost and market availability standpoint, is considered as one of the most promising FM replacers in aquafeed industry (Huang, Ding, Dai, & Ma, 2017). However, the high non‐digestible carbohydrates (NDCs) and anti‐nutritional factors (ANFs) in SBM decrease its nutritional value and thereby affecting fish growth (Montoya‐Camacho et al., 2018). Various heating treatments such as toasting, extrusion, and pelletizing have been used to inactive or eliminate the NDCs and ANFs from SBM. Among them, extrusion technology is commonly used to produce fish feeds, since physical properties (water stability, durability, hardness, oil absorption capacity, and buoyancy control) are improved. However, the single extrusion processing of SBM fails to remove NDCs and ANFs completely. Microbial fermentation can also eliminate these NDCs and ANFs, making SBM as a suitable proteinaceous aquafeed ingredient (Catalán, Villasante, Wacyk, Ramírez, & Romero, 2017). Recently, Obaroakpo, Iwanegbe, and Ojokoh (2016) reported lesser ANFs in the fermented‐extruded millet and soybean flour blend as compared with the extruded blend. Chi and Cho (2016) also reported the improved nutritional quality and bioactivity of the fermented SBM with different probiotics including *Lactobacillus*,* Saccharomyces,* and *Bacillus*, where the latter that is *Bacillus* sp. showed the best fermentative performance.

Another point noteworthy involves the rapid emergence of multi‐drug resistant pathogens that pose a significant burden on microbial infection management and public health globally (Thaya et al., 2016, 2018; Vaseeharan, Sivakamavalli, & Thaya, 2015). In this context, probiotics are one of the alternative approaches to antibiotics in aquaculture to prevent high mortality, inhibit pathogens (Vinoj, Vaseeharan, DavidJayaseelan, Rajakumaran, & Ravi, 2013; Vinoj, Vaseeharan, Thomas, Spiers, & Shanthi, 2014), and improve immune modulation (Elshaghabee, Rokana, Gulhane, Sharma, & Panwar, 2017). Generally, probiotics are applied in aquafeed after extrusion. However, fish farmers are facing redundant labor on using probiotic products due to the additional mixing step of probiotics in the formulated extruded pellet (EP) aquafeed. To limit this, feed manufacturers are looking for the heat‐resistant probiotics which can be directly included in aquafeed before extrusion. *Bacillus* spp. are widely used as probiotic supplements for fish with high heat stability and ability to survive in low pH (Elshaghabee et al., 2017). Additionally, they can also produce a variety of extracellular enzymes including amylase, protease, lipase, phytase, cellulase, and xylanase which can aid in feed digestion and nutrient absorption (Latorre et al., 2016). *Bacillus* probiotics are generally isolated from the animal's own gastrointestinal tract (GIT) (Mingmongkolchai & Panbangred, 2018; Vinoj et al., 2013). Therefore, in the present study, *B. licheniformis* was isolated from the intestine of olive flounder and screened for its probiotic potentials as well as heat stability in EP aquafeed.

## 2 MATERIALS AND METHODS

### 2.1 Isolation and identification

The indigenous intestinal microbes of olive flounder (*Paralichthys olivaceus*) were isolated using different nutrient media from BD™ Difco™ (U.S.), namely LB (Luria‐Bertani, pH 7.0 ± 0.2), MA (Marine agar, pH 7.6 ± 0.2), MRS (De Man, Rogosa and Sharpe, pH 6.5 ± 0.2), and R2A (Reasoner’s 2A agar, pH 7.2 ± 0.2), which were prepared in sea water (salinity 30 ± 3.0 psu). Seawater was procured from the local fish market, Seoul, Republic of Korea. The whole intestinal contents of sacrificed fishes (300–400 g) were procured from Aquafeed Research Center, National Institute of Fisheries Science (Pohang, Republic of Korea) and dissolved in sterile phosphate buffer saline (PBS, pH 7.4). The suitable diluted intestinal solution was spread on each agar plates and incubated at 25°C for 1 week. Single colonies with different morphologies were randomly picked, purified by streaking three times, and stored as glycerol stocks (40%, v/v) at −70℃ until use. The isolates were identified using 16S rDNA sequence analysis by a commercial service, Macrogen Inc. (Seoul, Republic of Korea). The genomic DNA of the isolates was extracted using an InstaGene™ Matrix (BIO‐RAD, USA). The region of 16S rRNA gene from the extracted DNA was amplified using universal oligonucleotide primers, forward 27F: 5′‐AGAGTTTGATCCTGGCTCAG‐3′ and reverse 1492R: 5′‐TACGGYTACCTTGTTACGACTT‐3′ primers. The amplicons were purified with a multiscreen filter plate (Millipore Corp., USA). The sequencing reaction of the purified PCR amplicons was performed using a PRISM BigDye(R) Terminator v3.1 Cycle sequencing kit (Applied Biosystem, USA) with forward 785F: 5′‐GGATTAGATACCCTGGTA‐3′ and reverse 907R: 5′‐CCGTCAATTCMTTTRAGTTT‐3′ primers and analyzed on ABI PRISM 3730XL DNA analyzer (Applied Biosystem) to yield about 1,300 bp sequences. The obtained gene sequences were then analyzed using GenBank Basic Local Alignment Search Tool (BLAST).

### 2.2 Screening of isolates

The identified 17 strains were screened for enzymatic and hemolytic activities. The strains were grown on agar plates corresponding to their isolated media containing 1% (w/v) of different substrates, namely soluble starch, carboxymethyl cellulose (CMC), and skim milk for the detection of amylase, cellulase, and protease, respectively, at 37°C for 48 hr. Spirit blue agar medium supplemented with tributyrin (2%, v/v) and tween 80 (0.3%, v/v) was used for the detection of lipase. The amylase and cellulase activities were confirmed by Gram’s iodine staining as described by Kasana, Salwan, Dhar, Dutt, and Gulati (2008). The lipase and protease production by the isolates were determined according to the clear zones of hydrolysis on the respective media following 48 hr of incubation at 37°C. All the strains were also checked for the hemolytic activity and determined by inoculating in blood agar (Himedia, Mumbai, India) plates containing 5% (w/v) of sterile sheep blood after 48 hr of incubation at 37°C (Angmo, Kumari, & Bhalla, 2016). The screened isolate *Bacillus licheniformis* SK3927 was submitted to Korean Culture Center of Microorganisms (KCCM) and designated as KCCM 43270. Antibiotic sensitivity of the KCCM 43270 against nine common antibiotics (ampicillin, oxacillin, cefepime, chloramphenicol, ciprofloxacin, clindamycin, gentamycin, tetracycline, and vancomycin) was determined by the disk diffusion method.

### 2.3 Degradative ability of KCCM 43270

The degradative ability of KCCM 43270 on complex nutritional sources, namely CMC, xylan from beechwood, and SBM was evaluated in the M9 medium. 1% (v/v) of overnight grown culture was inoculated into 100 ml M9 medium with 5% (w/v) of each CMC, xylan or SBM and incubated at 37°C and 120 rpm for 3 days. Samples were withdrawn at different time intervals and the viable cell count was measured by the drop plate method and expressed as log_10_ CFU/ml. The changes in reducing sugar (glucose equivalents) and protein content (bovine serum albumin, BSA equivalents) were determined using 3,5‐dinitrosalicylic acid (DNS) assay and bicinchoninic acid (BCA) assay (Pierce BCA protein assay kit; Thermo Scientific™, USA), respectively.

### 2.4 Adhesion ability of KCCM 43270

#### 2.4.1 Biofilm formation

The biofilm formation of KCCM 43270 was determined using crystal violet staining as described by Dawson, Valiente, Faulds‐Pain, Donahue, and Wren (2012) and Kalia, Prakash, Koul, and Ray (2017). About 1% (v/v) of overnight grown culture was inoculated into 5 ml sterile casein‐mannitol (CM) medium (casein digest 20 g/L and d‐mannitol 20 g/L). Then 200 μl of this CM medium was transferred into wells of a sterile 96‐well plate and incubated at 37°C for 48 hr. The medium was gently removed and stained with 25 μl of crystal violet (1%, w/v) for 15 min. After washing with distilled water twice, the dye was solubilized by adding 200 μl ethanol and the absorbance was measured at 600 nm using a microplate reader (Synergy 2; BioTek, USA) with ethanol as a blank.

#### 2.4.2 In vitro adhesion to mucus of olive flounder

The skin and intestinal mucus from olive flounder (body weight: 700–800 g) were procured from Sashimi shop (Seoul, Korea). The mucus was washed with sterile PBS (pH 7.4) several times and centrifuged with 12,000 *g* at 4°C for 5 min twice. The collected supernatant was adjusted to a final concentration of 0.5 mg/ml in PBS using a BCA assay kit. The prepared mucus (100 μl) was added into the wells of polystyrene microtiter plates and incubated at 4°C for overnight. After that, the wells were washed with PBS twice to remove the unbound mucus, followed by the addition of 100 μl overnight grown culture of KCCM 43270. After 1 hr of incubation at 30°C, the unbound cells were removed by PBS washing twice, the attached cells were re‐suspended in 50 μl of PBS and finally, the total viable cell count was measured. The adhesive ability (%) was calculated using the following equation:

$$\text{Adhesive}\;\text{ability}\;\left(\%\right)\;=\;\text{log}\;\text{CFU}\;N_{w}/\text{log}\;\text{CFU}\;N_{i}\;\times \;100\%$$

Where, *N*_w_ is the total viable cell count after washing and *N*_i_ is the initial viable cell count.

### 2.5 Acid, bile and heat tolerances of KCCM 43270 spores

The acid and bile tolerances of KCCM 43270 spores were determined according to Niu et al. (2018) with slight modifications. The spores were produced according to the method of Guo et al. (2016). About 1% (v/v) of overnight grown culture of KCCM 43270 was inoculated into sterile DSM medium (nutrient broth, 0.8% (w/v); KCl, 0.1% (w/v); MgSO_4_∙7H_2_O, 0.025% (w/v); Ca(NO_3_)_2_, 1 mmol/L; MnCl_2_, 10 μmol/L; FeSO_4_, 1 μmol/L) and incubated at 37°C and 200 rpm for 24 hr. Thereafter, the culture solution was subjected to heat (80°C for 20 min) and lysozyme (100 μg/ml for 10 min) treatments to kill the vegetative cells. The culture solution was centrifuged (4,000 *g*, 4°C, 10 min) and washed with PBS twice. The prepared spores (10^8^ CFU/ml) were re‐suspended in PBS (pH 2.5) or fresh LB broth with 0.3% (w/v) of bile salts for 3 hr. The heat resistance of spores was determined by the method of Guo et al. (2016). The spores (10^8^ CFU/ml) suspended in PBS were heated at 80, 90, 100, 110, and 120°C for different time intervals. At 100, 110, and 120°C, the heat resistance of the spores was also checked in SBM complex carrier (SBM:Egg shell powder fine particles; 5:2) with a moisture content of 25% (Lee et al., 2018). Acid, bile and heat resistance of spores were determined as viable cell count on LB agar plates and expressed as survival ratio by using following equation (Bao et al., 2010):

$$\text{Survival}\;\text{ratio}\;\text{(\textbackslash{}\% )}\;=\;\text{log}\;\text{CFU}\;N_{t}\;/\;\text{log}\;\text{CFU}\;N_{c}\;\times \;100\%$$

Where, *N*_t_ is the total viable count after treatment and *N*_c_ is the total viable count in control.

### 2.6 Application of KCCM 43270 spores in low‐fishmeal EP aquafeed

Low‐fishmeal aquafeed was manufactured at Aquafeed Research Center, National Institute of Fisheries Science (Pohang, Republic of Korea). The KCCM 43270 spores were immobilized in SBM complex carrier (SBM:Eggshell) and then mixed thoroughly with low‐fishmeal aquafeed to attain a final concentration of 10^6^ CFU/g. The mixed feed was then subjected to twin‐screw extruder (ATX‐II, Fesco Precision Co., Daegu, South Korea) with the following conditions: feeder supply speed, 70 kg/hr; conditioner temperature, 80°C; <5 min; barrel temperature, 120–130°C; <1 min; main screw speed, 650 rpm. Thereafter, low‐fishmeal EP feed was air‐dried at 60°C for 3 hr followed by vacuum fish oil coating and stored at −20°C until use. At each step of feed processing, the spore count was quantified by measuring the viable cell count (CFU/g). For this, an aliquot (10 g) of samples were withdrawn, heated at 80°C for 20 min to remove other contaminants, serially diluted, and plated on LB agar plates.

### 2.7 Statistical analysis

The data were expressed as the mean ± standard deviation (*SD*) from three replicates. Mean values were compared by one‐way analysis of variance (ANOVA) procedures employing the Duncan test at *p* < 0.05, using statistical software SPSS (ver. 24, USA).

## 3 RESULTS AND DISCUSSION

### 3.1 Isolation, identification, and characterization

To establish potential probiotic additives which can be directly provided in low‐fishmeal EP aquafeeds, we have isolated and identified 17 intestinal microbes from olive flounder (Table 1). All the identified strains were further screened for their enzymatic and hemolytic activities. Only two strains, *Staphylococcus epidermis* SK4045 and *B. licheniformis* SK3927 (KCCM 43270) displayed multi‐enzymatic and non‐hemolytic activities. Being an opportunistic human pathogen, *S. epidermidis* was not considered for further study (Vuong & Otto, 2002). *B. licheniformis* is a widely used probiotic additive with benefits of improved growth performance, feed utilization, immune response, gut health, and infection resistance (Deng, Dong, Tong, & Zhang, 2012; Sadat Hoseini Madani, Adorian, Ghafari Farsani, & Hoseinifar, 2018). In the present study, KCCM 43270 was susceptible to the nine used antibiotics, except oxacillin and clindamycin, implying a low risk of antibiotic‐resistant gene transfer (Supporting Information, Table S1). The resistance to clindamycin is considered as an intrinsic property of *B. licheniformis*, conferring a desirable trait for recovering the intestine microbiota of the host after antibiotic treatment (Jeong, Jeong, & Lee, 2017). Considering these properties, *B. licheniformis* KCCM 43270 was selected and further characterized for putative probiotic properties.

**Table 1:** Identification and characterization of microbes isolated from intestine of olive flounders

Stock #	Isolation	Identification			Enzyme activity[Link]				Hem[Link]
	Media	Description	Query coverage (%)	Identity (%)	A	C	L	P	
SK3917	MRS	*Staphylococcus warneri*	57	99	—	—	++	—	γ
SK3921	MA	*Micrococcus luteus*	99	99	—	—	—	+	α
SK3927	LB	*Bacillus licheniformis*	99	99	+	+++	+	+	γ
SK3928	LB	*Edwardsiella tarda*	100	99	—	—	—	—	γ
SK3929	LB	*Ochrobactrum pseudogrignonense*	99	100	—	—	+	+	γ
SK3939	LB	*Micrococcus lylae*	98	99	—	—	+	+	α
SK3941	LB	*Halomonas* sp.	99	99	—	—	—	—	γ
SK3957	LB	*Streptococcus parauberis*	99	99	—	—	—	—	γ
SK4030	R2A	*Bacillus cereus*	100	99	—	—	+	+++	α
SK4031	R2A	*Staphylococcus hominis*	99	99	—	—	+	—	γ
SK4036	R2A	*Ponticoccus gilvus*	98	96	—	—	+	++	γ
SK4038	R2A	*Staphylococcus capitis*	99	99	—	—	—	—	γ
SK4039	R2A	*Janibacter* sp.	85	99	—	—	+	±	α
SK4040	R2A	*Staphylococcus saprophyticus*	99	99	—	—	+	±	γ
SK4042	R2A	*Dermacoccus nishinomiyaensis*	99	99	±	—	+	++	γ
SK4045	R2A	*Staphylococcus epidermidis*	99	99	+	+	+	++	γ
SK4047	MA	*Corynebacterium pilbarense*	99	98	—	—	—	—	γ

Enzyme activity: A, Amylase; C, CMCase; L, Lipase; P, Protease. —，no activity; +, weak activity; ++, moderate activity; +++, strong activity.

Hem: Hemolysis (α, complete hemolysis; γ, non‐hemolysis).

### 3.2 Enzyme activities and degradative ability of KCCM 43270

Supplementation of hydrolytic enzymes in animal feed is an important approach to improve the digestion and absorption of poorly available nutrients or to remove ANFs (Kiarie, Romero, & Nyachoti, 2013). Therefore, the enzyme production by KCCM 43270 was further examined (Supporting Information, Table S2). It exhibited alkaline phosphatase, esterase (C4), esterase lipase (C8), acid phosphatase, naphtol‐AS‐BI‐phosphohydrolase, similar to other previously reported *Bacillus* strains (Lee et al., 2017). Additionally, the isolate also showed glucosidase and β‐galactosidase activity. However, the isolate displayed no activity for the carcinogenic enzyme, β‐glucuronidase.

**Figure 1:**
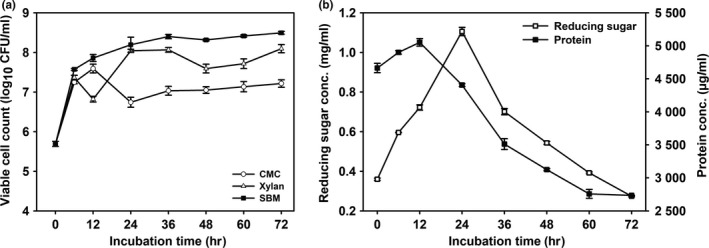
Degradative ability of *Bacillus licheniformis* KCCM 43270. (a) Growth in M9 medium containing carboxymethyl cellulose (CMC), xylan and SBM; (b) Change in reducing sugar and protein contents in M9 medium containing soybean meal (SBM)

Furthermore, the growth ability of KCCM 43270 was evaluated in CMC, xylan, or SBM as sole carbon source in the M9 medium (Figure 1a). The isolate was able to grow in all the complex nutritional sources. The growth was attributed to the different enzymatic activities of the isolate. *Bacillus* spp. have been reported earlier to produce diverse carbohydrate and protein degrading enzymes such as amylase, arabinase, cellulase, chitinase, dextranase, galactanase, glucanase, mannanase, xylanase, protease, lipase, and others (Elshaghabee et al., 2017). The maximum growth was observed in SBM (Figure 1a) with the concomitant change in reducing sugar and protein concentration (Figure 1b). In the first 24 hr, the reducing sugar content increased sharply and thereafter decreased. A similar trend was observed for the protein concentration after 12 hr. This might be due to the consumption of simple sugars and peptides for maintaining the cell growth further.

### 3.3 Adhesion ability of KCCM 43270

**Figure 2:**
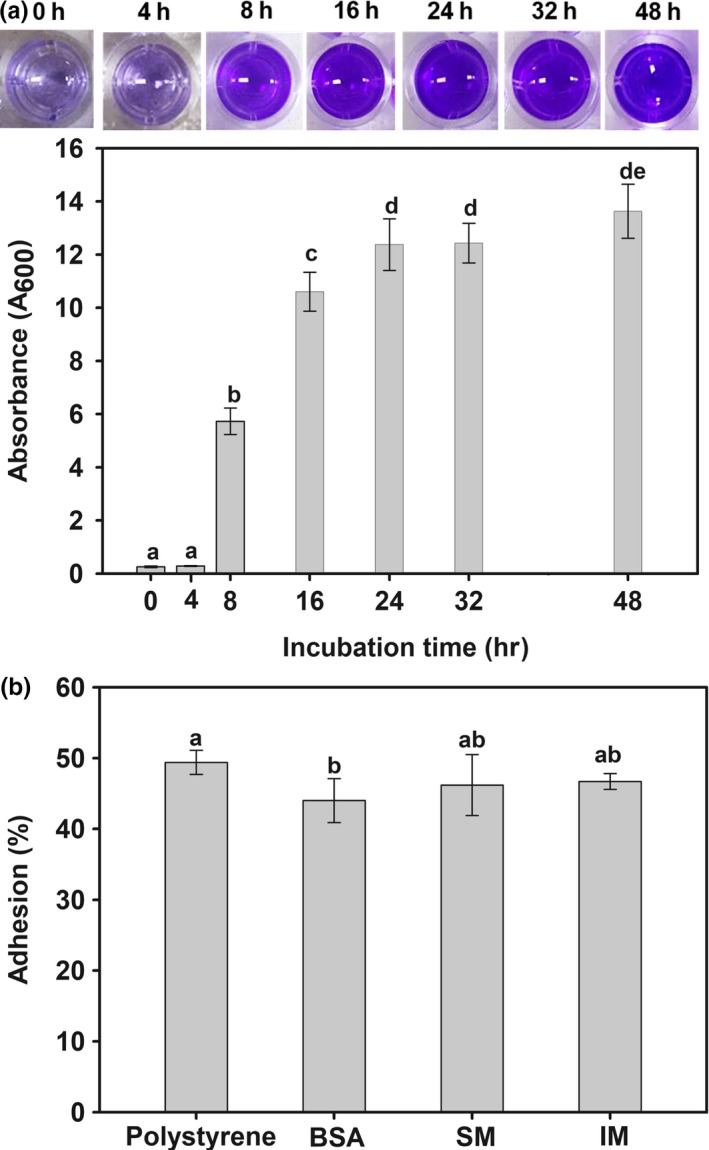
Adhesive ability of *Bacillus licheniformis* KCCM 43270. (a) Biofilm formation; (b) Cell adhesion, BSA: Bovine serum albumin; IM: Intestine mucus; SM: Skin mucus

Biofilm, a beneficial trait for the colonization in the host GIT, is a complex microbial community encased in a self‐produced extracellular matrix (Fang, Jin, & Hong, 2018). The KCCM 43270 was able to produce biofilm at high levels (OD_595_ 10–12) following 16 hr of incubation (Figure 2a). This can be attributed to the high sporulation ability (>90% at 24 hr) of the isolate (Supporting Information, Figure S1) as previously described by Hong et al. (2009). Biofilm formation by the isolate might enhance adherence to the host surfaces and microbial aggregation as well as resistance to stressful conditions.

Adhesion to the mucus is another important criterion for the selection of probiotics. The adhesion ability of the isolate to intestinal mucus (IM), skin mucus (SM), and polystyrene was 46.2%, 46.7%, 49.7%, respectively (Figure 2b). The adhesion to polystyrene can be correlated with cell surface hydrophobicity (CSF), the high CSF is an indicator of good adhesion (Balcázar et al., 2007). In this study, the isolate exhibited ability of biofilm formation, adhesion to mucus, or polystyrene reflecting the potential of *B. licheniformis* to colonize in the GIT of fish.

### 3.4 Acid, bile, and heat tolerances of KCCM 43270 spores

**Figure 3:**
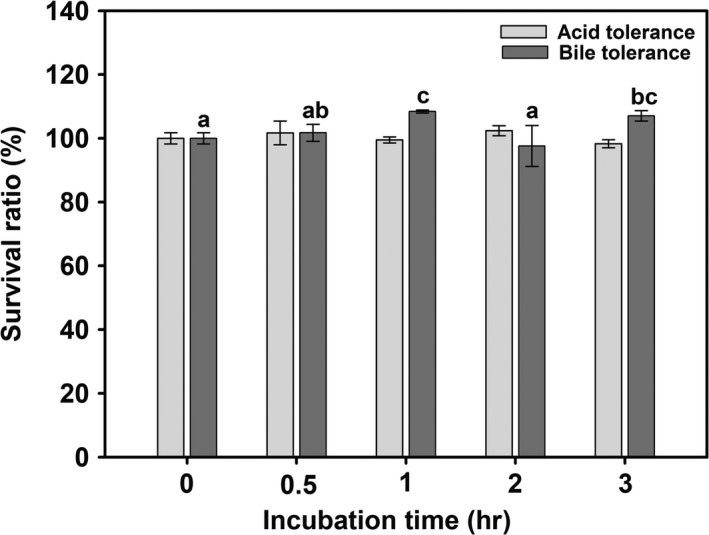
Acid and bile tolerances of *Bacillus licheniformis* KCCM 43270 spores

**Figure 4:**
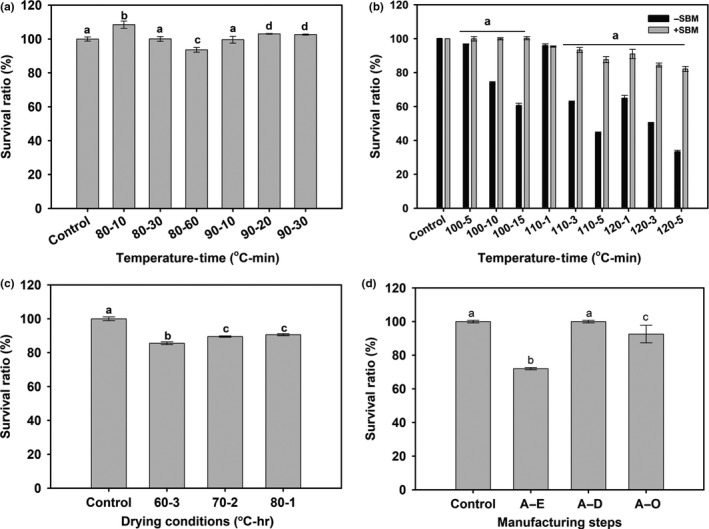
Heat resistance of *Bacillus licheniformis* KCCM 43270 spores. (a) 80–90°C; (b) 100–120°C with or without soybean meal (SBM) complex carrier; (c) Drying at 60–80°C with SBM complex carrier; (d) EP feed processing (Control: After‐mixing with raw ingredients, A–E: After‐extrusion, A–D: After‐drying, A–O: After‐oil coating)

As probiotics are orally administered, tolerance to acid in stomach and bile in the proximal intestine is an important criterion for selecting potential probiotic isolates to ensure their viability and functionality. The *Bacillus* spores can survive in acidic conditions, tolerate bile salts, and other hostile conditions of GIT. Additionally, the *Bacillus* spores are more stable during processing and storage of food and pharmaceutical preparations, which make them more suitable ingredients for health‐promoting formulations (Elshaghabee et al., 2017). In the present study, the spores of KCCM 43270 exhibited complete tolerance to acid (pH 2.5) and bile (0.3%, w/v) stress following 3 hr of incubation (Figure 3) and thereby indicating their probiotic potential. Manufacturing of aquafeeds often involves heating at different steps for better efficiency in terms of storage, digestibility, and shelf‐life. The high temperature may reduce the viability of probiotic strains in the aquafeed. Therefore, the heat resistance of KCCM 43270 spores was evaluated from 80 to 120°C. The spores showed complete survival at 80°C for 60 min and 90°C for 30 min (Figure 4a). However, at 100, 110, and 120°C, the spores exhibited survival only for 5, 1, and <1 min, respectively, and thereafter decreased in a time‐dependent manner. Therefore, the spore viability above 90°C was further achieved through blending with SBM complex carrier. As shown in Figure 4b,c, after blending with the carrier, the viable spore count was not reduced, only 1 log CFU decrease was observed even at 120°C. Additionally, the probiotic blend displayed only 1 log CFU decrease at different drying conditions. The high survival rate of KCCM 43270 spores at high temperatures suggests its use as EP aquafeed additive.

### 3.5 Application of KCCM 43270 spores in low‐fishmeal EP aquafeed

In this work, the survival ability of KCCM 43270 in low‐fishmeal EP feed was evaluated by adding KCCM 43270 spores into raw feed ingredients prior to the extrusion process (Figure 4d). Following the extrusion, the log CFU was decreased by only 18% and then remained virtually constant. This can be explained by the fact that during extrusion, the moisture content was high around 30%, and after drying, it was reduced to <8%; and thereby the difference in log CFU was potentially affected by the dry matter content. To the best of authors’ knowledge, it is reported for the first time that *B. licheniformis* KCCM 43270 spores can survive the extrusion process during the manufacture of low‐fishmeal EP aquafeed.

## 4 CONCLUSIONS

In conclusion, *B. licheniformis* KCCM 43270 isolated from olive flounder intestine has potential probiotic characteristics in relation to antibiotic resistance, enzyme production, degradative ability, sporulation, biofilm formation, adhesions to intestinal and skin mucus, and stress tolerance (low pH, bile, and heat). Besides, the isolate survived the extrusion process during low‐fishmeal EP aquafeed manufacturing and thereby could also be developed as a heat‐resistant aquafeed additive.

## CONFLICT OF INTEREST

The authors declare no conflict of interests.

## AUTHORS CONTRIBUTION

K.M. Niu and D. Kothari are the co‐first author. K.M. Niu conducted the experiments, analyzed the data, and drafted the manuscript. D. Kothari analyzed the data, wrote and revised the manuscript. W.‐D. Lee and J.M. Lim assisted in the experiments. S. Khosravi and S.‐M. Lee assisted in the aquafeed data analysis. B.‐J. Lee, K.‐W. Kim, and H.‐S. Han collected the intestinal content form the fishes and manufactured the extruded feed. S.‐K. Kim as a supervisor designed and funded the whole study. All authors read and approved the final manuscript.

## ETHICS STATEMENT

The intestinal contents of fishes were collected by Aquafeed Research Center, National Institute of Fisheries Science (Pohang, South Korea) following the guidelines of the Animal Ethics Committee Regulations (2016‐NIFS‐IACUC‐06).

## Data Availability

The strain *Bacillus licheniformis* was deposited in Korea Culture Center of Microorganisms (KCCM), Republic of Korea with the accession number of KCCM 43270. All data generated or analyzed during this study are included in this published article.
