# Supplementary material for: Autochthonous Bacillus licheniformis: Probiotic potential and survival ability in low‐fishmeal extruded pellet aquafeed
Source: Microbiologyopen. 2018 Nov 16;8(6):e00767. doi: 10.1002/mbo3.767 (PMC6562133; doi:10.1002/mbo3.767)
Supplement: Supplementary file 1 [file MBO3-8-e00767-s001.docx]

*Supporting information*

**Figure legend**

**Figure S1** The sporulation ability of *B. licheniformis* KCCM 43270 in DSM medium.

**
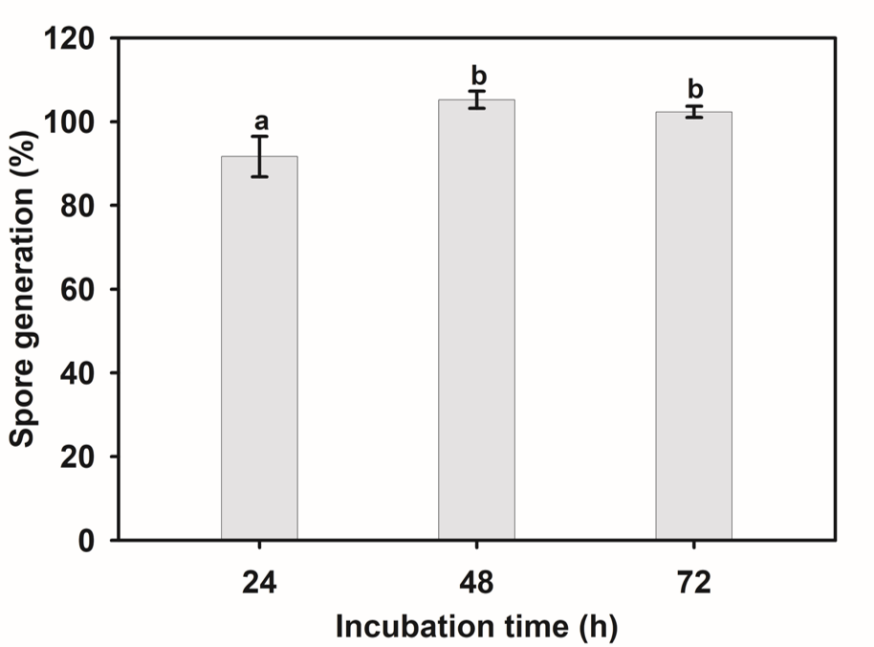
**

**Figure S1**

**Table S1** Antibiotic susceptibility of *B. licheniformis* KCCM 43270**.**

| Antibiotic | Class | Con, ug/disc | Clear zone diameter, mm |
| --- | --- | --- | --- |
| Amp | Penicillins | 10 | 15.0 ± 0.0 |
| Oxa | Oxacillin | 1 | - |
| Fep | Cephalosporins | 30 | 13.7 ± 0.6 |
| Chl | Chloramphenicol | 30 | 16.3 ± 0.6 |
| Cpx | Fluoroquinolones | 5 | 31.0 ± 1.0 |
| Cli | Lincomycin | 2 | - |
| Gen | Aminoglycosides | 10 | 17.0 ± 0.0 |
| Tet | Tetracyclines | 30 | 28.3 ± 0.6 |
| Van | Glycopeptide | 30 | 18.3 ± 0.6 |

Amp - Ampicillin, Oxa - Oxacillin, Fep - Cefepime,

Chl - Chloramphenicol, Cpx - Ciprofloxacin, Cli - Clindamycin,

Gen - Gentamycin, Tet - Tetracycline, Van – Vancomycin.

**Table S2** Exogenous enzymatic activities of *B. licheniformis* KCCM 43270 using APIzym kit.

| No. | Enzyme | Activity |
| --- | --- | --- |
| 1 | Control | **-** |
| 2 | Alkaline phosphatase | **+** |
| 3 | Esterase (C4) | **+** |
| 4 | Esterase Lipase (C8) | **+** |
| 5 | Lipase (C14) | **-** |
| 6 | Leucine arylamidase | **+** |
| 7 | Valine arylamidase | **-** |
| 8 | Crystine arylamidase | **-** |
| 9 | Trypsin | **-** |
| 10 | a-chymotrypsin | **+** |
| 11 | Acid phosphatase | **+** |
| 12 | Naphtol-AS-BI-phosphohydrolase | **+** |
| 13 | a - galactosidase | **-** |
| 14 | b - galactosidase | **+** |
| 15 | b - glucuronidase | **-** |
| 16 | a - glucosidase | **+** |
| 17 | b - glucosidase | **+** |
| 18 | N –acetyl - b - glucosaminidase | **-** |
| 19 | a - mannosidase | **-** |
| 20 | a - fucosidase | **-** |

+: positive activity; -: negative activity
